# Evolution of impedance values in cochlear implant patients after early switch-on

**DOI:** 10.1371/journal.pone.0246545

**Published:** 2021-02-19

**Authors:** Jeff Jia-Fu Wei, Tao-Hsin Tung, Lieber Po-Hung Li

**Affiliations:** 1 Department of Otolaryngology, Cheng Hsin General Hospital, Taipei, Taiwan; 2 Department of Medical Research and Education, Cheng Hsin General Hospital, Taipei, Taiwan; 3 Faculty of Medicine, School of Medicine, National Yang-Ming University, Taipei, Taiwan; 4 Department of Medical Research, China Medical University Hospital, China Medical University, Taichung, Taiwan; 5 Department of Speech Language Pathology and Audiology, College of Health Technology, National Taipei University of Nursing and Health Sciences, Taipei, Taiwan; Medical University Hannover; Cluster of Excellence Hearing4all, GERMANY

## Abstract

Cochlear implantation is currently the most effective treatment modality for severe to profound sensorineural hearing loss. Over the past few years, at the Department of Otolaryngology, Cheng Hsin General Hospital (Taipei, Taiwan), cochlear implant devices have been switched on within 24 hours of their implantation. Differences in impedance evolution after early switch-on for different devices have not been previously discussed. The present study aimed to investigate the impedance evolution of one device and the factors influencing this after early activation. Results are compared to published results of other devices. A total of 16 patients who received Advanced Bionics^TM^ devices and had early activation within 24 hours of implantation, were included in the study. Impedance telemetry was recorded intraoperatively and postoperatively at 1 day, 1 week, 2 weeks, 4 weeks and 8 weeks. A stepwise increase was observed in the impedance evolution. To the best of our knowledge, the present study is the first to investigate the impedance evolution of the different devices after early switch-on within 24 hours of implantation and its influencing factors. Further research with a longitudinal design to compare the differences in electrode impedances between patients activated early versus those activated after a few weeks will be necessary for the disclosure of the underlying mechanisms.

## Introduction

Cochlear implantation is currently the most effective treatment modality for severe to profound sensorineural hearing loss, not only in aging populations but also in children. Previous studies by the authors demonstrated that hearing, speech, and music performance were improved after implantation [1, 2]. The surgical techniques for cochlear implantation and postoperative management have improved in recent decades. In our department, a minimally invasive approach has been routinely used, which made the initial switch-on of the device possible within 24 hours post-implantation [3]. The safety and feasibility of initial switch-on within 24 hours after cochlear implantation was proved in one of our researches [4]. One of the benefits of early switch-on is that it can help subjects return to normal activity and life as soon as possible, and with immediate rehabilitation programs, subjects can notice the improvements and better adapt to their new device.

Among the various measurement modalities available for cochlear implantation, impedance telemetry is currently the most widely used, due to its ability to provide the electrode integrity, current flow, and power consumption of implants [5]. The association between impedance change and the surrounding environment have been reported in previous studies [5–7]. The advantages of electrode impedance are that it gives information about the stability of the device and the performance of the cochlear implant, and thus allows for adaption of the clinical stimulus levels and settings.

An investigation of one of the major manufactures’ device has been conducted by the authors, which revealed a significant drop in impedance within 24 hours after cochlear implantation [8]. A longitudinal study consecutively showed a significant rise of impedance in all channels one week post-operatively, and the impedance then behaved differently in the different segments of cochlea [9]. However, the impedance in every segment began to be relatively stable 4 weeks after surgery [9]. On the contrary, we found that the impedance of another manufacture’s device (Advanced Bionics^TM^, Westinghouse, Pl, USA) increased significantly within 24 hours after the surgery [10]. However, the long-term variation of impedance for the device remained unknown.

In the present study, the charts of 16 patients were reviewed. They all received the surgery of cochlear implantation with the same kind of device. We aimed to demonstrate the scenario in impedance evolution for the cochlear implant after switch-on within 24 hours post-operatively and the possible contributing factors.

## Methods

### Ethics statement

The study conformed to the Declaration of Helsinki and the guidelines of the Institutional Ethics and Research Committee of the Cheng-Hsin General Hospital, which approved the study. The requirement for informed consent was waived.

### Participants

A total of 16 subjects who underwent cochlear implantation (8 males and 8 females; 2 to 75 years old, mean age = 43.25 years) in Cheng Hsin General Hospital were recruited into the current study. They all used Mid-Scale electrode arrays (length: 15 mm; type of contacts: planar) of the manufacturer Advanced Bionics^TM^. Of the 16 subjects, 11 received implantation on the left side and 5 received implantation on the right. All of the devices were initially switched on within 24 hours after the surgery.

### Surgical procedure

A minimally invasive surgical technique was used with a small postauricular incision (2.5-3cm), followed by minimal mastoidectomy for harvesting of a cortex bone chip. Cold instruments were used as far as possible, then electrocauterization, massive irrigation of the surgical field, and hyaluronic acid gel coverage of the round window pre- and post-implantation. After the insertion, harvested bone chip was covered at the defects of the mastoid cavity. Postoperative X-rays were conducted for every subject to confirm full insertion of the electrode array and full insertion of the electrode array was confirmed for all patients. A more detailed surgical procedure has been described in our previous studies [4, 8].

### Impedance measurements

Impedance values were measured intraoperatively, 24 hours after the operation (1D), 1 week postoperatively (1W), 2 weeks postoperatively (2W), 4 weeks postoperatively (4W), and 8 weeks postoperatively (8W). The impedance measurement was performed in the monopolar (MP) mode, with a pulse width of 72 μs, and a current level of 32 μA. For convenience in the analysis and assessment, we defined apical electrodes as CH 1 to CH 5, middle electrodes as CH 6 to CH 10, and basal electrodes as CH 11 to CH 16.

### Statistical analysis

All statistical analyses were performed using SPSS version 18.0.0 (SPSS, Inc., Chicago, IL, US). A paired sample t-test was used to compare values from consecutive fitting sessions. Continuous data were presented as the mean ± standard deviation (SD). Statistical significance was set at P<0.05.

### Results (Tables 1 and 2, Figs 1 and 2)

**Fig 1:**
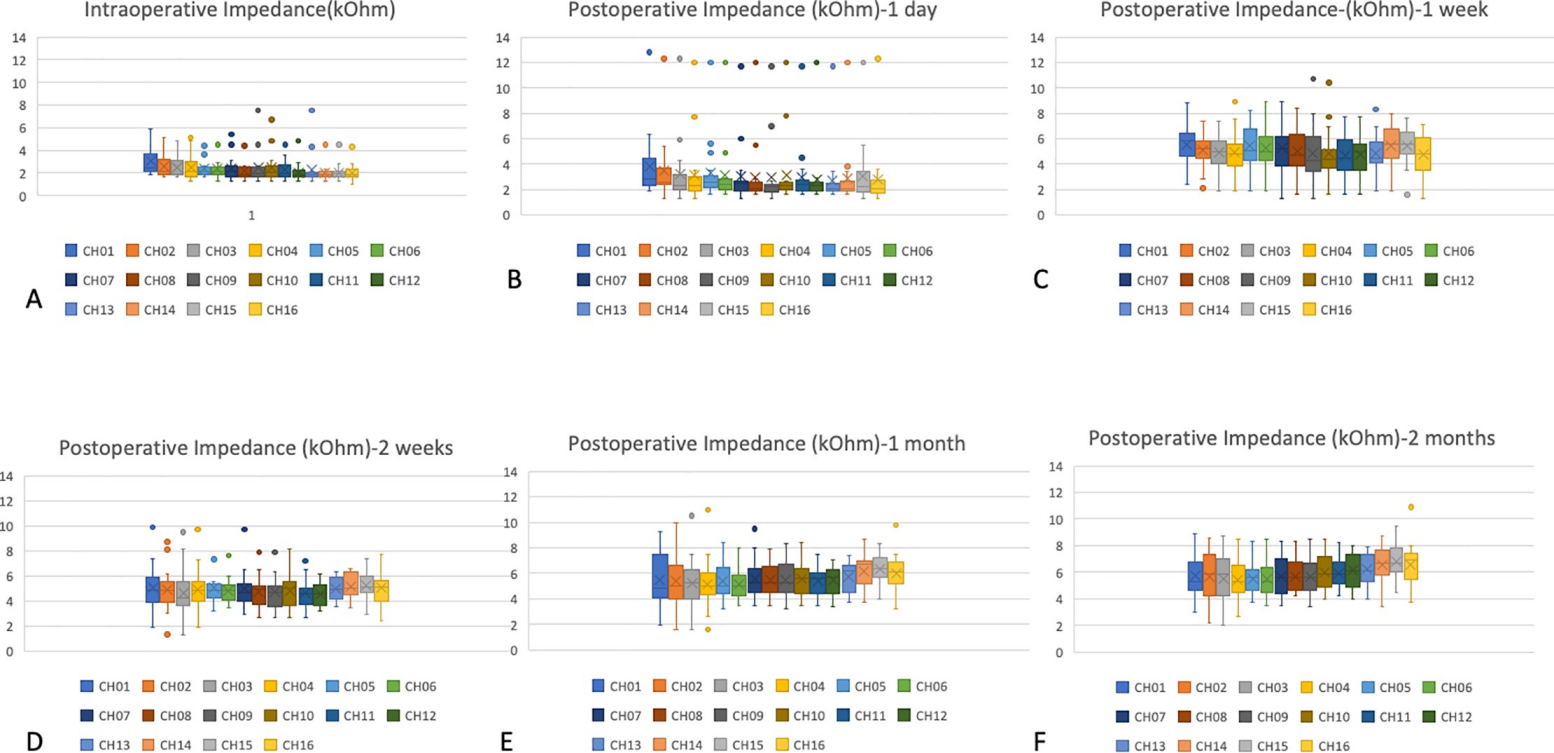
Impedance measurements for each of the 16 electrodes. Median values are displayed as horizontal lines and mean values as crosses. Circles represent extreme values. (A) Intraoperative measurements, (B) 1 day after surgery, (C) 1 week after surgery, (D) 2 weeks after surgery, (E) 4 weeks after surgery, (F) 8 weeks after surgery.

**Fig 2:**
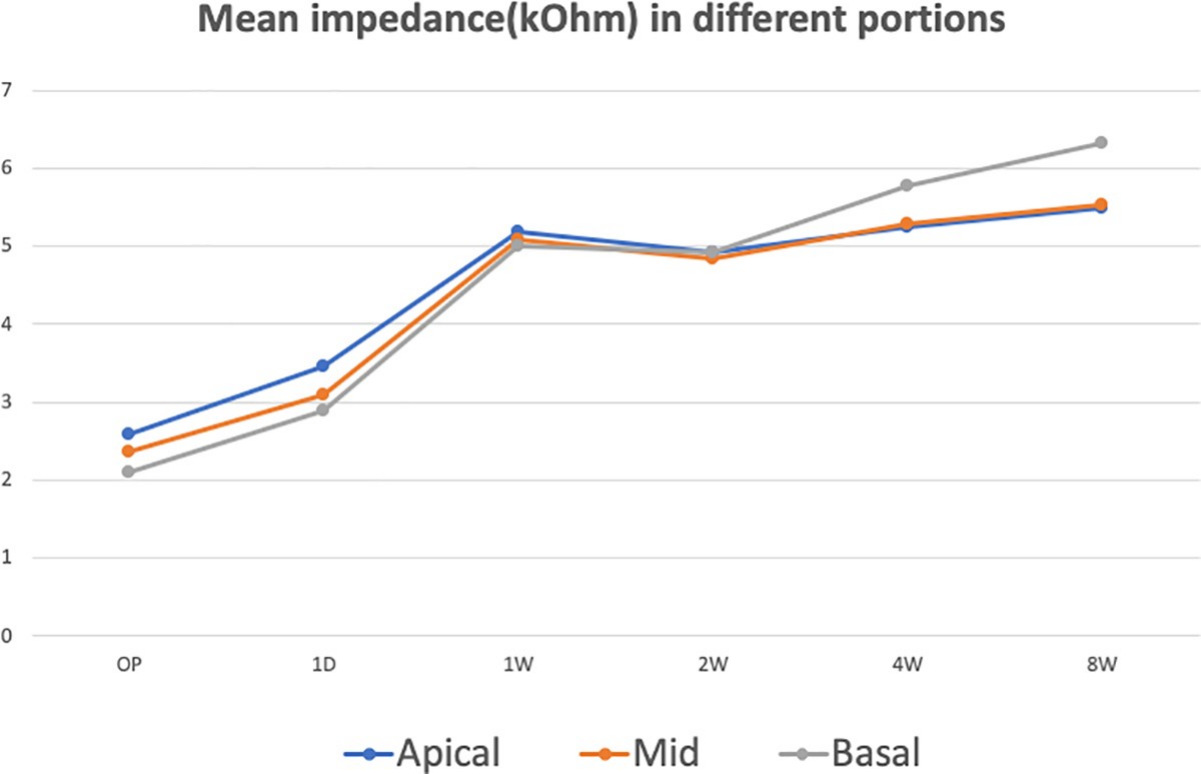
Evolution of the mean impedance for the apical, middle and basal electrodes over 8 weeks.

**Table 1:** Mean impedance values (kOhm) in each channel.

		OP	1D	1W	2W	4W	8W
**Apical electrodes**	CH01	3.07	3.94	5.47	5.09	5.48	5.71
	CH02	2.56	3.49	5.05	4.86	5.32	5.63
	CH03	2.43	3.26	4.99	4.68	5.15	5.46
	CH04	2.51	3.25	4.97	4.90	5.08	5.26
	CH05	2.39	3.31	5.48	5.06	5.23	5.34
	Mean	2.59	3.45	5.19	4.92	5.25	5.48
	S.D.	0.28	0.29	0.26	0.17	0.15	0.19
**Middle electrodes**	CH06	2.35	3.21	5.29	4.76	4.93	5.29
	CH07	2.38	3.12	5.28	5.03	5.44	5.53
	CH08	2.22	2.98	5.04	4.81	5.36	5.53
	CH09	2.42	2.96	4.90	4.76	5.26	5.46
	CH10	2.49	3.17	4.91	4.85	5.42	5.83
	Mean	2.37	3.09	5.09	4.84	5.28	5.53
	S.D.	0.09	0.11	0.19	0.11	0.21	0.19
**Basal electrodes**	CH11	2.24	3.06	4.75	4.64	5.23	5.86
	CH12	2.03	2.81	4.81	4.63	5.43	6.08
	CH13	2.26	2.78	4.88	4.93	5.73	6.19
	CH14	2.01	2.93	5.46	5.14	6.10	6.52
	CH15	2.02	3.02	5.38	5.18	6.20	6.71
	CH16	1.99	2.78	4.69	4.99	5.99	6.52
	Mean	2.09	2.89	4.99	4.92	5.78	6.31
	S.D.	0.13	0.13	0.34	0.24	0.39	0.32

**Table 2:** Impedance changes between the different timepoints.

		OP vs. 1D	1D vs. 1W	1W vs. 2W	2W vs. 4W	4W vs. 8W
**Apical electrodes**	CH1	0.072	0.049*	0.805	0.173	0.443
	CH2	0.066	0.043*	0.400	0.061	0.221
	CH3	0.089	0.028*	0.746	0.025*	0.141
	CH4	0.128	0.023*	0.438	0.293	0.445
	CH5	0.072	0.010*	0.648	0.308	0.638
**Middle electrodes**	CH6	0.096	0.013*	0.658	0.341	0.066
	CH7	0.108	0.011*	0.550	0.029*	0.727
	CH8	0.105	0.011*	0.464	0.007*	0.484
	CH9	0.249	0.014*	0.361	0.015*	0.488
	CH10	0.150	0.019*’	0.419	0.004*	0.044*
**Basal electrodes**	CH11	0.089	0.024*	0.548	0.003*	0.001*
	CH12	0.096	0.015*	0.536	<0.001*	0.002*
	CH13	0.383	0.012*	0.233	<0.001*	0.023*
	CH14	0.059	0.005*	0.660	<0.001*	0.069
	CH15	0.050	0.006*	0.687	<0.001*	0.078
	CH16	0.136	0.015*	0.047*	<0.001*	0.041*

*Statistical significance was defined as P<0.05.

At our institution, we used a minimally invasive approach for cochlear implant surgery. It was therefore possible for us to activate the cochlear implants within 24 hours of the surgery. All patients received a thorough examination prior to the initial activation to ensure it was safe to proceed. There were no reported post-operative complications, such as hematoma, wound infection, or flap failure. In addition, no short or open circuits were noted for the devices intra- or post-operatively. The impedance values were measured intraoperatively, 1D, 1W, 2W, 4W, and 8W post-operatively.

Compared with the intraoperative measurements, the impedance at 1D was not significantly different. At 1W, the impedance telemetry demonstrated a significant rise in all channels compared with 1D (CH1, p = 0.049; CH6, p = 0.013; CH11, p = 0.024; CH16, p = 0.015). There were no significant differences between the impedance of 1 W and 2 W. The only significant impedance increase was in CH 16. Then the impedance values significantly increased at 4W and this increase was statistically significant for CH3 and from CH7 to CH16 (p = 0.025 at CH3, 0.029 at CH7, 0.003 at CH11, and <0.001 at CH16). At 8W, the increasing trend of the impedance became smooth in the apical and middle electrodes but persisted in the basal electrodes.

## Discussion

In this study, we observed the evolution of the impedance changes in patients who received Advanced Bionics^TM^ Mid-Scala cochlear implants. There were some differences between the findings of this present study and the author’s previous studies, which investigated the Nucleus 24RECA implant system. To the best of our knowledge, this is the first research yet disclosing results about the long-term variations of impedance after initial switch-on within 24 hours subsequent to cochlear implantation in human beings using Advanced Bionics^TM^ system.

### Intraoperative measurements

The intraoperative impedance telemetry revealed that the apical electrodes had the highest values and that the basal electrodes had the lowest values (Table 1), which was typically seen at implantation. This was also consistent with one of our previous studies about different implant device [9]. Although the underlying mechanism of this finding was unclear, the reason for this should be the different contact size. The size of the contact area is smaller distally (0.5 X 0.5 mm for the contact #1) and larger proximally (0.7 X 0.7 mm for the contact #16) for the device in the present study. Since the impedance values would be inversely related to the geometric surface area of the electrode according to the spherical model [5], the impedance could thus be expected to be lower at the basal turn.

Also surprising is that the intraoperative values of impedance with the mid-scala electrode are that low. It is therefore reasonable to speculate that some kind of initial activation has taken place before the measurement. The recommendations about the electrode conditioning on measuring intraoperative impedance has been routinely followed in our institute. Otherwise, we can make sure that the device in the present study was not in use including NRI or other measurements involving electrical stimulation before the measurement. Additionally, Advanced Bionics devices were known to measure electrode impedances somewhere between 4 to 6 -μs after the initiation of the cathodic phase of the biphasic cathodic-anodic pulse according to the measurement algorithm. The influence of measurement 4 to 6 -μs after the initiation of the cathodic phase could have on the impedance values would be that the value consisted only of “access resistance” without the component of “polarization impedance” [5].

### Impedance within 24 hours after the implantation

The difference is not significant between the impedance measured within 24 hours after the implantation and that measured intra-operatively in the present study (Tables 1 and 2). This was different to one study of the same system by the authors [10]. Our previous study was focused on patients who received HiFocus^TM^ 1-J electrode array implantations and the patients in the present study received HiFocus^TM^ Mid-Scala implants. After implantation, a Mid-Scala array lies closer to the modiolus than a 1-J array. Besides, less trauma would be caused by the Mid-Scala electrodes with the round window approach. The above two reasons could explain the stable impedance on day one. One might wonder that a 1-J array will have more space to develop fibrosis and sheath formation [11, 12], which in turn could lead to the sheath formation effect [6, 13] and thus a significant increase in impedance as observed in our previous study [10]. However, a complete sheath can’t be expected to form at day one despite cell cover begins within hours of implantation [5, 7]. Therefore, the space around the electrode carrier might influence impedance values later on but very unlikely on day 1.

### Stepwise increase from 24 hours to 1 month

The stepwise increase in impedance was one of the novel findings of the present study (Fig 2). The first increase appeared at 1W. Then, the impedance difference was not significant between 1 W and 2 W. The impedance continued to have a significant increase between 2W and 4W, but it’s noteworthy that the difference was insignificant within apical contacts except for CH3. Our hypothesis for the above-mentioned scenario of the impedance value was that it might be due to changes in local fibrosis and the relief of the acute inflammation. Intracochlear fibrosis starts 2 to 5 days after implantation, which could account for the first increase [9]. In general, relief of the acute inflammation can explain the transient trend of impedance decrease at 1W. Since the fibrosis will go on but also begin to resolve [9], the impedance increased again on a smooth basis between 2W and 4W.

There were differences between the present study and our previous study which focused on devices by Coclear^TM^ [9]. In the current study, the round window approach was used. With the Cochlear device, a cochleostomy was drilled. These different approaches have an influence on the tissue formation within scala tympani at least in guinea pigs (i.e. less tissue with the round window approach), and therefore most likely also on the impedances [14]. Although we cannot directly compare our data to the data from the previous study [9], the differences in impedance evolution might be accounted for at least partly by the different surgical approaches.

Also, Advanced Bionics devices measure electrode impedances somewhere between 4 to 6 -μs after the initiation of the cathodic phase of the biphasic cathodic-anodic pulse. This primarily represents “access resistance”. Cochlear devices measure electrode impedances at the end of the cathodic phase of the biphasic cathodic-anodic pulse. This represents total impedance which is a combination of access resistance and polarization impedance. Access resistance is known to slowly increase over time after activation, whereas polarization impedance is known to decrease over time [5]. The two cochlear implant manufacturers measure electrode impedances at different point during the cathodic phase, which in turn could very likely be one of the major reasons for the differences in the evolution of electrode impedances.

### Slowdown of the impedance increases from 1 to 2 months

The second increase was followed by a slowdown, except for in the basal electrodes (Fig 2). The impedance in the basal electrodes continued to increase during this period. A more intense fibrotic change in the basal electrodes has been reported in several previous studies [15, 16]. The fibrosis and new bone formation were furthermore found to be focused at the regions of basal turn corresponding to the sites of trauma on the lateral wall due to implant insertion [17]. Despite the results from the previous studies [15–17] were incomparable to those of the present study due to different techniques of surgery, there were implications which might be extrapolated to our findings. With a cochleostomy a huge trauma is caused basally especially at the lateral wall. With the round window insertion this is avoided and the first major contact with the lateral wall is expected much later at the end of the relatively straight part of the basal turn. In spite of the differences in the areas of damage resulting from surgical approaches, the mechanism(s) for our findings of a steeper increase in impedance for the basal turn could at least in part be ascribed to the severer degree of fibrosis and new bone formation in comparison to other sections of cochlea.

## Conclusion

The impedance of the Mid-Scale electrode arrays was found to be basically increased since initial activation 24 hours within the implantation all the way to 8 weeks after the surgery before the variation became smooth. To the best of our knowledge, our research is the first to report on the stepwise increasing impedance in electrodes of Advanced Bionics^TM^ devices after early activation within 24 hours post-operatively. We also discuss the difference in impedance evolution of different cochlear implant devices. However, whether the early activation play a major role in the evolution of electrode impedances or not remained to be elucidated. Further research with a longitudinal design to compare the differences in electrode impedances between patients activated early versus those activated after a few weeks will be necessary for the disclosure of the underlying mechanisms.
